# Comorbidity of Alcoholism and Anxiety Disorders: The Role of Family Studies

**Published:** 1996

**Authors:** Kathleen R. Merikangas, Denise Stevens, Brenda Fenton

**Affiliations:** Kathleen R. Merikangas, Ph.D., is the director of the Genetic Epidemiology Research Unit in the Departments of Epidemiology and Psychiatry at Yale University School of Medicine, New Haven, Connecticut. Denise Stevens, Ph.D., is an associate research scientist and Brenda Fenton, M.Sc., is a research associate in the Department of Epidemiology at Yale University School of Medicine, New Haven, Connecticut

**Keywords:** comorbidity, AOD dependence, anxiety state, family study, prevalence, hereditary factors, risk factors, gender differences, treatment

## Abstract

People with alcoholism frequently also suffer from an anxiety disorder. The mechanisms underlying this comorbidity remain unclear. Clinical findings indicate that anxiety disorders may lead to the development of alcoholism. Conversely, alcoholism may contribute to the development of anxiety symptoms. Family studies have reported elevated rates of anxiety disorders in the relatives of patients with alcoholism and vice versa, suggesting that both disorders may share some susceptibility factors. The Yale Family Study of the comorbidity of alcoholism and anxiety confirmed these observations. The study also found gender-specific differences in the risk for some comorbid anxiety disorders. Moreover, the relatives of people with alcohol dependence or anxiety were at increased risk for alcohol dependence but not alcohol abuse.

Numerous clinical and epidemiological studies^1^ have demonstrated the comorbidity of alcoholism^2^ and anxiety disorders (reviewed in Wesner 1990; Kushner et al. 1990; George et al. 1990; Crowley and Riggs 1995; Schuckit and Hesselbrock 1994). The confirmation of an association between alcoholism and anxiety disorders in large-scale epidemiological studies suggests that the observed frequent comorbidity is not attributable to the fact that patients with both disorders may be more likely to enter treatment and thus be overrepresented in clinical studies (Regier et al. 1990*a*; Kessler et al. 1996). The mechanisms underlying this comorbidity remain unknown, largely because of the highly variable manifestations (i.e., the heterogeneity) of both disorders and the disparate methodologies employed in the various studies. In general, however, the comorbidity of alcoholism and anxiety appears to be far more common in women than in men, in individuals diagnosed with alcohol dependence rather than alcohol abuse, and among people with phobic states rather than panic or generalized anxiety states (for a definition of these terms, see the following section).

This article provides an overview of the prevalence and the potential mechanisms underlying the comorbidity of alcoholism and anxiety disorders. The article also describes how family studies may help elucidate the association between these disorders and presents data from a recent family study conducted at Yale University.

## Anxiety Disorders

The anxiety disorders of adulthood compose one of the most common groups of psychiatric disorders in the general population, with prevalence rates in the community as high as 25 percent (Kessler et al. 1994). Anxiety is medically defined as “the apprehension of danger and dread accompanied by restlessness, tension, tachycardia^3^ and dyspnea^4^ unattached to a clearly identified stimulus” (Stedman 1996, p.187). Several different subtypes of anxiety exist that are defined by differences in the specific stimulus (or lack thereof) and the nature of the anxiety. The American Psychiatric Association’s *Diagnostic and Statistical Manual of Mental Disorders, Fourth Edition* (DSM–IV) specifies 12 major categories of anxiety disorders, such as panic disorder with or without agoraphobia, specific or social phobia, generalized anxiety disorder, or substance-induced anxiety disorder.

Some of the more common anxiety disorders and their symptoms include the following:

- Panic disorders with or without agoraphobia are characterized by panic attacks—sudden episodes of intense fearfulness or terror that are accompanied by autonomic nervous system reactions, such as palpitations (i.e., a pounding or racing of the heart), sweating, or shortness of breath.
- Agoraphobia with and without accompanying panic disorder involves the avoidance of places or situations from which escape might be difficult—for example, being in a crowd of people, or traveling on a bridge or in an elevator.
- Phobias are characterized by specific fears of objects or situations, such as spiders and heights (i.e., specific phobia) or speaking in public (i.e., social phobia).

### Prevalence of Co-Occurring Alcoholism and Anxiety Disorders

Clinical studies reveal that 23 to 70 percent of patients in alcoholism treatment also suffer from anxiety disorders, particularly anxiety neurosis and phobias (Kushner et al. 1990; Merikangas and Angst 1995). Conversely, 20 to 45 percent of patients with anxiety disorders have histories of alcoholism (Kushner et al. 1990). Much of the variability in the comorbidity rates of anxiety disorders and alcoholism can be attributed to differences in the definitions of anxiety employed in the studies, the period for which the prevalence was determined, and the groups of alcoholics studied. For example, more people have experienced comorbid anxiety and alcoholism at some time in their lives than at the time they are actually studied (Merikangas et al. 1996*a*). Moreover, comorbid anxiety disorders occur more commonly among people diagnosed with alcohol dependence than among those diagnosed with alcohol abuse (Helzer and Pryzbeck 1988; Regier et al. 1990*b*; Kessler et al. 1996).

### Mechanisms for Comorbidity

At least two possible mechanisms exist that could explain the frequent co-occurrence of alcoholism and anxiety disorders: (1) one disorder leads to the development of the other disorder (i.e., a causal relationship exists between the two disorders) and (2) both disorders may be caused by the same underlying factor(s) (i.e., both disorders have a common etiology) and thus represent different manifestations or stages of the same disease.

Most clinical data favor the first hypothesis and suggest that anxiety disorders lead to alcoholism because people self-administer many antianxiety agents, most commonly alcohol, to relieve symptoms of anxiety (Lader 1972). Moreover, many patients with anxiety have used alcohol deliberately to cope in fear-inducing situations (Smail et al. 1984; Stockwell et al. 1984). In nationwide surveys, high school students also have reported reduction in anxiety or tension as a major reason for adolescent drug use (Johnston et al. 1986). Finally, in many people, anxiety symptoms precede the onset of alcoholism, often by several years, suggesting that alcoholism is secondary to anxiety disorders (Stockwell et al. 1984; Weiss and Rosenberg 1985; Merikangas et al. 1994). Anxiety may play a role in initiating as well as in maintaining alcoholism (Stockwell et al. 1984).

Conversely, alcoholism also may contribute to the development of anxiety symptoms. For example, anxiety is a common symptom of alcohol withdrawal (Schuckit and Monteiro 1988). In addition, prolonged alcohol consumption leads to a marked deterioration of the expression of emotions and feelings (i.e., affect) that is characterized by anxiety and depression (Mendelson and Mello 1966; Nathan et al. 1971). In some people, alcohol also may induce cardiovascular symptoms (e.g., palpitations, increased heart rate, or sweating) that mimic anxiety or heighten awareness of physical symptoms (Mello and Mendelson 1979).

The role of alcoholism in inducing anxiety was supported by findings that phobias and panic attacks did not persist in alcoholic men who were assessed during hospitalization and after 1 year of followup (Penick et al. 1988). Thus, the timing of assessment appears to be critical when determining the comorbidity of alcoholism and anxiety disorders. The reliable assessment of co-occurring alcoholism and specific anxiety disorders also is hampered by the fact that in clinical studies, which mostly include male subjects, the numbers of alcoholics with anxiety disorders are small compared with the numbers of alcoholics with other comorbid psychiatric disorders, such as antisocial personality or other drug abuse.

## The Role of Family Studies in Analyzing Comorbidity of Alcoholism and Anxiety Disorders

Family studies compare the prevalence rates of certain disorders among the relatives of subjects (i.e., probands) with the disorders to the relatives of probands without the disorders. Family studies may help discriminate between alternative mechanisms for the co-occurrence of two or more disorders by examining the transmission of pure (e.g., alcoholism only) and combined (e.g., alcoholism plus anxiety) forms of the disorders among the relatives of probands with pure and comorbid disorders (Merikangas and Gelernter 1990). For example, family studies have been employed to investigate the mechanisms underlying the comorbidity of alcoholism, anxiety, and depression (Merikangas et al. 1994).

If two disorders have a causal relationship, the relatives of probands with the causal disorder will manifest an increased risk for the pure form of the causal disorder and for the combination of both disorders, but not for the pure form of the comorbid disorder. For example, if anxiety caused alcoholism, relatives of probands with anxiety should have an increased rate of pure anxiety or anxiety combined with alcoholism, but should have normal rates of pure alcoholism.

If the two disorders share a common etiology (i.e., similar underlying biochemical defects or detrimental environmental factors lead to the development of either disorder), then the relatives of probands with the pure form of one disorder also will exhibit elevated rates of the pure form of the comorbid disorder compared with those of the general population. Consequently, if alcoholism and anxiety shared a common etiology, the relatives of probands with pure anxiety also should be at increased risk for pure alcoholism and vice versa.

To discriminate between these alternatives, family studies must include sufficient numbers of probands with pure forms of each disorder as well as of control probands with neither disorder. Using appropriate sampling and diagnostic procedures, one then can estimate differences in the rates of the disorders among the relatives of affected probands and the relatives of the control probands. When analyzing these data, however, it is important to exclude factors that may lead to overestimates of comorbidity. These factors include, for example, the following: the exclusive use of clinical samples, which may overestimate comorbidity because patients with two disorders are more likely to seek treatment; overlaps in the diagnostic criteria of the disorders studied; and confounding factors, such as gender-specific differences in the comorbidity of certain disorders.

### Findings of Previous Family Studies of Alcoholism and Anxiety

Numerous studies have demonstrated that both alcoholism (Merikangas and Gelernter 1990; McGue 1994) and anxiety disorders (Cohen et al. 1951; Noyes et al. 1978; Crowe et al. 1983; Fyer et al. 1993) run in families. Family studies of both alcoholism and anxiety disorders also have reported elevated rates of the other condition among the probands’ relatives. For example, first-degree relatives of probands with anxiety disorders exhibited a significantly increased risk for alcoholism (Cohen et al. 1951; Noyes et al. 1978; Munjack and Moss 1981; Harris et al. 1983; Leckman et al. 1983; Maier et al. 1993*a*). Likewise, anxiety rates were elevated among the relatives of alcoholic probands (Merikangas et al. 1985; Maier and Merikangas 1996). These studies, however, generally did not relate the rates of the comorbid disorders among the relatives to the presence or absence of comorbidity in the probands and therefore could not determine whether the disorders were inherited independently of each other.

Only a few family studies have examined specifically the patterns of cotransmission of alcoholism and anxiety disorders among first-degree relatives of affected probands (Maier et al. 1993*a*; Maier and Merikangas 1996). Maier and Merikangas (1996) investigated the comorbidity between affective disorders (e.g., depression), anxiety disorders, and alcoholism, including the patterns of cotransmission of alcoholism and panic disorder. The study’s findings suggested that shared susceptibility factors exist for alcoholism and panic disorder, because the relatives of probands with pure panic disorder also exhibited an elevated risk of alcoholism. Similarly, a recent uncontrolled family study of anxiety disorders suggested an etiologic relationship between anxiety disorders and alcohol and other drug abuse (Skre et al. 1994).

Another study that simultaneously examined the comorbidity and cotransmission of alcoholism, anxiety, and depression has yielded evidence that both alcoholism and anxiety can be transmitted independently within families and that comorbid alcoholism and anxiety can be cotransmitted (Merikangas et al. 1994). The study did not allow conclusions about the mechanisms underlying cotransmission, however, because it did not include any probands who exhibited pure alcoholism or pure anxiety, and all the alcoholic subjects suffered from secondary alcoholism (i.e., alcoholism caused by depression). Accordingly, another study—the Yale Family Study—was conducted that included probands with pure alcoholism, pure anxiety disorders, or both disorders and which was designed specifically to investigate the patterns of familial transmission of the comorbidity of alcoholism and anxiety disorders (Merikangas et al. 1996*b*). Findings from this study are presented in the following sections.

## The Yale Family Study of the Comorbidity of Alcoholism and Anxiety

### Sample Characteristics

The Yale Family Study included 226 probands, including patients with alcoholism and/or anxiety disorders and normal control subjects. The patients were recruited from outpatient specialty clinics at the Connecticut Mental Health Center in New Haven, Connecticut. All patients treated at the clinics over a 3-year period were screened for eligibility in the study, and those who met the appropriate diagnostic criteria were invited to participate. The control subjects were randomly selected from the population of the greater New Haven area. All probands were assigned to one of four lifetime diagnostic groups based on their predominant psychopathology as follows:

- Forty-two probands met the criteria of the *Diagnostic and Statistical Manual of Mental Disorders, Third Edition, Revised* (DSM–III–R) for alcohol dependence.
- Seventy-six probands met the DSM–III–R criteria for an anxiety disorder.
- Forty-seven probands fulfilled the DSM–III–R criteria for both alcohol dependence and an anxiety disorder.
- Sixty-one normal control subjects had no history of a DSM–III–R axis I disorder (i.e., a major psychiatric disorder, such as an affective disorder, an anxiety disorder, substance abuse or dependence, or antisocial personality).

The presence or absence of alcoholism and anxiety disorders in the probands’ first-degree relatives and spouses was assessed through semi-structured diagnostic interviews. Family history information on all families also was obtained using semistructured diagnostic interviews.

### Prevalence of Alcoholism and Anxiety in Relatives

The relatives of probands who were alcohol dependent and/or had an anxiety disorder were at an increased risk for developing alcohol dependence and/or an anxiety disorder themselves (table 1). For example, whereas 4 percent of the relatives of the control probands developed alcohol dependence, the rate among relatives of alcohol-dependent probands was 15 percent. Similarly, the rate of anxiety disorders was 13 percent among relatives of control probands but 21 percent among relatives of probands with anxiety disorders. The rates of alcohol dependence and anxiety disorders also were increased in the relatives of probands with comorbid alcohol dependence and anxiety disorders. Finally, the presence of anxiety disorders in the probands slightly increased the risk for alcohol dependence in their relatives, whereas alcohol dependence in the probands did not increase their relatives’ risk for anxiety disorders. Similarly, Maier and colleagues (1993*b*) demonstrated an increased risk of alcoholism in probands with panic disorder, but not the reverse.

These findings suggest some role for shared etiologic factors in the comorbidity of alcoholism and anxiety disorders that could account for the increased risk of alcoholism in relatives of probands with anxiety disorders. These shared etiologic factors could include genetic factors predisposing to both types of disorders, biological environmental risk factors, nonbiological environmental factors (e.g., a disruptive family environment or parental abuse or neglect), or exposure to prenatal environmental factors (e.g., maternal alcohol use). The manifestation of a particular disorder could then be determined by the timing of the exposure to the risk factors and/or the influence of additional genetic or environmental factors. Findings from a study of female twins indicate that common genetic factors may underlie both alcoholism and panic disorder at least to some extent (Kendler et al. 1995).

To analyze further the comorbidity of alcoholism and anxiety disorders in the relatives of all probands, calculations (i.e., odds ratios) of alcohol-dependent male and female relatives were computed for developing specific anxiety disorders as well as other psychiatric disorders (table 2). These odds ratios indicate whether alcohol dependence and other disorders are associated by comparing the proportion of relatives concordant for the two disorders (i.e., who have either both disorders or none of them) to the proportion of relatives discordant for the two disorders (i.e., who experience either alcohol dependence without anxiety or anxiety without alcohol dependence).

The analyses demonstrated that alcohol-dependent male relatives were about twice as likely to exhibit an anxiety disorder as nonalcohol-dependent male relatives, whereas for female alcohol-dependent relatives, the likelihood was increased 3.7 times. These findings indicate that in addition to a person’s family history, other factors, such as gender, influence the risk for comorbid disorders. For certain anxiety disorders, the gender differences were even more significant. For example, the odds ratio for comorbid panic disorder was 0.6 for alcohol-dependent male relatives but 4.2 for alcohol-dependent female relatives. Similar gender-specific differences existed for some comorbid affective disorders, such as major depression and bipolar disorder.

### Comorbidity and Cotransmission of Alcohol Dependence Versus Alcohol Abuse

Using complex statistical analyses that took into consideration the probands’ disorders, the relatives’ disorders, and other factors including the relatives’ ages, gender, and interview status (e.g., direct interview versus family history information), the Yale Family Study also compared the risk ratios^5^ for the development of alcohol abuse and alcohol dependence in the relatives. The results reveal that alcohol dependence in the probands was more strongly associated with alcohol dependence than with alcohol abuse in the relatives (table 3). For example, 24 percent of the relatives of alcohol-dependent probands met the lifetime criteria for alcohol dependence compared with only 7 percent of the relatives of the control subjects, yielding a risk ratio of approximately 3. In contrast, the differences in alcohol abuse rates among the relatives of the alcohol-dependent probands (10 percent) and the relatives of the control subjects (7 percent) were not significant. In general, the risk of alcoholism was higher in male relatives than in female relatives and lower in older relatives than in younger ones (i.e., in earlier birth cohorts). These observations confirm the results of a previous study demonstrating significantly greater heritability of alcohol dependence than of alcohol abuse (Pickens et al. 1991). Furthermore, they validate the distinction between alcohol abuse and dependence introduced by Edwards and Gross (1976) and implemented in the DSM–III–R and other subsequent diagnostic schemes.

Anxiety disorders in the probands also were significantly associated with alcohol dependence (adjusted risk ratio of 1.46) but not alcohol abuse (adjusted risk ratio of 0.99) in the relatives (table 3). Thus, anxiety disorders in the probands conveyed an increased risk of alcohol dependence in their relatives even after controlling for the familial transmission of alcoholism and other risk factors, including sex, age, and interview status of the relatives. These findings support the hypothesis mentioned previously that alcohol dependence and anxiety disorders may result, at least in part, from shared underlying risk factors. Analyses of a sample of female twins revealed that the shared liability for anxiety disorders and alcoholism is partially attributable to common genetic risk factors (Kendler et al. 1995).

## Conclusions

### Associations Between Alcoholism and Anxiety

The results of the Yale Family Study confirmed the well-established familial aggregation of both anxiety disorders and alcoholism as well as the common comorbidity between both types of disorders. The frequency of the observed comorbidity of alcoholism and other major psychiatric disorders also was consistent with the results of previous clinical and epidemiological studies in which alcoholism was strongly associated with antisocial personality disorder, affective disorders, and anxiety disorders. These findings illustrate the importance of using family studies to investigate the mechanisms underlying comorbidity. Compared with other experimental approaches, family studies examining high-risk relatives (i.e., relatives of probands with a specific diagnosis) remove some of the selection biases associated with clinical studies and increase the number of potential subjects with the desired comorbidity. Thus, family studies can produce more stable estimates of the risk of comorbidity.

The Yale Family Study also found evidence of gender differences in the comorbidity between alcoholism and specific anxiety disorders. Previous studies had found higher general psychiatric comorbidity in women (Angst et al. 1990) but had not systematically addressed gender differences in the comorbidity of alcoholism and anxiety. Moreover, these studies frequently had yielded inconsistent results regarding gender-specific comorbidity (Smail et al. 1984; Chambless et al. 1987), in part because many clinical studies were limited to male alcoholics or included only small sample sizes, resulting in nonrepresentative comorbidity rates.

### Implications for Diagnosis, Treatment, and Prevention

The findings regarding the comorbidity and cotransmission of alcoholism and anxiety disorders have important implications for the diagnostic classification, treatment, and prevention of these disorders. For example, both the Yale Family Study and the investigation by Pickens and colleagues (1991) underscored the significance of the distinction between alcohol abuse and alcohol dependence used in current diagnostic classification systems. Both studies demonstrated that alcohol dependence was strongly transmissible in families, whereas alcohol abuse was not. Accordingly, investigators should pay attention to the diagnostic status of their subjects, and research or clinical studies that include both probands with alcohol abuse and with alcohol dependence may not be able to detect certain relationships between alcoholism and other related factors or disorders. For example, studies of the associations between alcoholism and biological markers should focus on subjects who are alcohol dependent, whereas studies of the role of environmental factors in alcoholism development could perhaps best be addressed using subjects diagnosed with alcohol abuse.

The comorbidity of alcoholism and anxiety disorders also may affect the evaluation and treatment of individuals with these conditions, irrespective of the disorder for which these people seek treatment. For example, alcoholics with comorbid anxiety experience more severe alcohol withdrawal and an increased tendency to relapse (Johnston et al. 1991; LaBounty et al. 1992). Conversely, alcoholism resulting from self-medication with alcohol often goes unrecognized in treatment settings for anxiety disorders. In fact, alcohol withdrawal can closely mimic the symptoms of panic and generalized anxiety. Furthermore, persons with comorbid anxiety and alcoholism often manifest additional comorbid disorders, particularly affective disorders. Consequently, when designing long-term treatment strategies, one must evaluate potential manifestations of anxiety and depression during alcohol withdrawal and subsequent abstinence (Anthenelli and Schuckit 1993).

The relationships between alcoholism and anxiety disorders described in this article also provide key information for both primary and secondary prevention of these disorders. For example, family histories of these conditions can serve as important indicators of an increased risk for the disorders in the offspring of these families. Moreover, the existence of a potential etiological pathway from anxiety disorders to alcoholism suggests that children experiencing high levels of anxiety symptoms or anxiety disorders have a heightened risk for using alcohol to self-medicate anxiety symptoms, particularly in the presence of other known risk factors for alcoholism. The early identification and treatment of these children could help prevent potential subsequent alcohol abuse and dependence.

## Figures and Tables

**Table 1:** Comorbidity of Alcohol Dependence and Anxiety in Subjects (i.e., Probands) and Their Relatives Participating in the Yale Family Study

	Diagnoses of Probands			

	Alcohol Dependence and Anxiety Disorders	Alcohol Dependence	Anxiety Disorders	Normal/Other
Number of Relatives	221	162	357	293
Diagnoses of Relatives				
Alcohol dependence and anxiety disorders (%)	5	7	6	3
Alcohol dependence (%)	20	15	8	4
Anxiety disorders (%)	19	11	21	13
Normal/other (%)	55	66	65	80

**Table 2:** Adjusted Odds Ratios^1^ of the Comorbidity of Psychiatric Disorders in Alcohol-Dependent Male and Female Relatives of Subjects (i.e., Probands) in the Yale Family Study

Comorbid Disorders in Relatives^2^	Adjusted Odds Ratios for Risk of Comorbidity for Relatives of Probands From the Yale Family Study	

	Alcohol-Dependent Male Relatives	Alcohol-Dependent Female Relatives
**Anxiety (all)**	2.0 (1.2–3.3)^3^**	3.7 (1.9–7.0)***
Panic disorder	0.6 (0.1–5.2)	4.2 (1.8–9.8)***
Generalized anxiety	2.3 (1.1–4.5)*	2.8 (1.4–5.7)**
Social phobia	1.7 (0.8–3.4)	4.1 (2.0–8.6)***
Agoraphobia	—	2.4 (0.9–6.2)
**Affective**		
Major depression	5.5 (3.1–9.6)***	2.1 (1.1–4.3)*
Bipolar disorder	2.4 (0.4–14.6)	9.6 (2.8–33.4)***
Dysthymia^4^	3.7 (1.8–7.6)***	3.4 (1.7–7.0)***
**Antisocial personality**	34.5 (7.9–151.7)***	36.8 (9.0–149.8)***

1 The adjusted odds ratios are calculated by comparing the proportion of relatives who are concordant for the two disorders (i.e., have both alcoholism and another disorder or have none of these disorders) with the proportion of relatives who are discordant for the two disorders (i.e., have either alcoholism and no other disorder or vice versa).

2 These diagnoses are not mutually exclusive.

3 Numbers in parentheses indicate 95-percent confidence intervals.

4 A type of depression that tends to occur in elderly persons with debilitating physical disorders, multiple interpersonal losses, and chronic marital difficulties.

Significance level:

* *p* < 0.05;

** *p* < 0.01;

*** *p* < 0.001.

**Table 3:** Adjusted Risk Ratios^1^ for the Comorbidity and Cotransmission of Alcohol Dependence or Alcohol Abuse and Anxiety Disorders Among Subjects (i.e., Probands) and Their Relatives in the Yale Family Study

Disorder in Probands	Adjusted Risk Ratio of Alcohol Dependence or Abuse in Relatives of Probands	

	Alcohol Dependence	Alcohol Abuse
Alcohol dependence	2.83***	1.61+
Anxiety	1.46*	0.99

1 Similar to odds ratios, risk ratios provide a measure of the association between two disorders by comparing the risks for alcohol dependence or alcohol abuse in relatives of probands with alcohol dependence or anxiety to the risks for relatives of probands without alcohol dependence or anxiety.

Significance level:

+ *p* < 0.1;

* *p* < 0.05;

*** *p* < 0.001.

## Footnotes

1 Clinical studies use relatively small numbers of patients with the disorder under investigation as their subjects (e.g., alcoholics or people with an anxiety disorder who are undergoing treatment). Therefore, all subjects of a clinical study share certain characteristics. Epidemiological studies, in contrast, include large numbers of subjects who are drawn from the general population. Thus, they include people with and without the disorder under investigation who have not been selected on the basis of specific clinical characteristics.

2 The term “alcoholism” as used in this article encompasses the diagnoses of both alcohol abuse and alcohol dependence as defined in the American Psychiatric Association’s *Diagnostic and Statistical Manual of Mental Disorders, Fourth Edition*.

3 Tachycardia means “increased heart rate.”

4 Dyspnea means “shortness of breath or difficulty in breathing.”

5 Similar to odds ratios, risk ratios provide a measure for the strength of the association between two disorders.
